# A 34-Week Size Uterus with a Complete Hydatidiform Mole: Hook Effect and Severe Anemia with No Vaginal Bleeding

**DOI:** 10.1155/2018/8201949

**Published:** 2018-02-13

**Authors:** Rodney McLaren, Vijaya Bayya, Mohamad Irani

**Affiliations:** ^1^Department of Obstetrics and Gynecology, Maimonides Medical Center, Brooklyn, NY 11219, USA; ^2^Ronald O. Perelman and Claudia Cohen Center for Reproductive Medicine, Weill Cornell Medical Center, New York, NY 10021, USA

## Abstract

Complete hydatidiform mole is an abnormal pregnancy that usually presents with vaginal bleeding and markedly elevated serum ß-hCG levels. We report a rare case of complete hydatidiform mole occurring in a 16-year-old nulligravid who presented with a 34-week size uterus and a relatively low serum ß-hCG level (722 IU/L)—likely related to the “hook effect”—and severe anemia (hemoglobin: 6.1 g/dL) despite the absence of vaginal bleeding. She also reported right flank pain and was diagnosed with moderate right hydronephrosis owing to the compression exerted by the enlarged uterus on the right ureter. The patient received a total of 6 units of packed red blood cells and was managed by dilation and evacuation followed by serial monitoring of serum ß-hCG levels. Therefore, complete mole can present with symptoms related to an enlarged uterus and severe anemia before the occurrence of vaginal bleeding. It is also important to note that a negative urine pregnancy test or relatively low serum ß-hCG level should prompt repeating the measurement on diluted sample to prevent the “hook effect.”

## 1. Introduction

A hydatidiform mole is an abnormal pregnancy that is characterized by trophoblastic proliferation and villous edema within the placenta [1]. There are two distinct entities of molar pregnancy: partial and complete mole. Partial moles are characterized by the presence of fetal or embryonic tissues, chorionic villi of different sizes featured by their focal trophoblastic hyperplasia and focal swelling, and marked villous scalloping [2]. However, complete moles are characterized by the absence of embryonic or fetal tissues, diffuse trophoblastic hyperplasia and hydropic swelling of villi, and marked atypia of trophoblast at the implantation site [2].

The majority of complete moles present with vaginal bleeding and markedly elevated ß-hCG values [3, 4]. We report a rare case of complete molar pregnancy who presented with an enlarged uterus and symptoms related to the compression on adjacent organs, severe anemia despite the absence of vaginal bleeding, and a relatively low serum ß-hCG level owing to the “hook effect.”

## 2. Case Presentation

**Figure 1:**
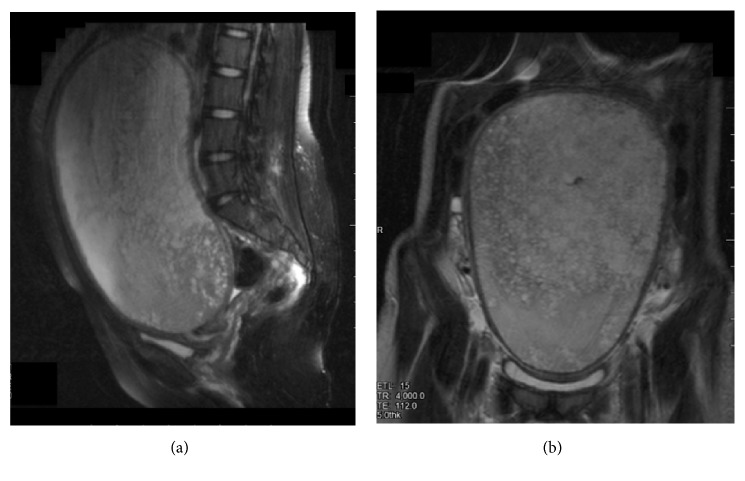
Sagittal (a) and coronal (b) views of an abdominal-pelvic Magnetic Resonance Imaging showing an enlarged uterus (24 × 11 × 17 cm) with a large intrauterine heterogeneous mass.

**Figure 2:**
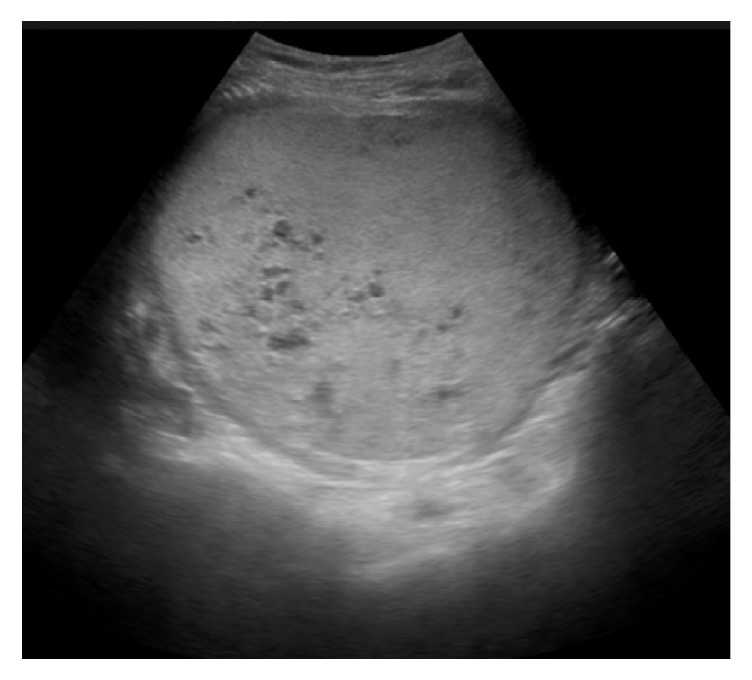
A pelvic ultrasound showing a large intrauterine heterogeneous mass that includes multiple discrete anechoic spaces consistent with complete mole.

A 16-year-old nulligravid who presented 37 days after her last menstrual period to the emergency department with a new onset diffuse abdominal pain, abdominal distention, and right flank pain. She also reported urinary frequency that started two days prior to presentation. The patient denies vaginal bleeding, fever, or chills. Her medical, surgical, and social histories were negative. She did not have any family history of uterine, colon, ovarian, or breast cancer. She was afebrile and normotensive but tachycardic (125 beats/min). Her abdomen was distended with a nontender 34-week size uterus. The initial blood work revealed severe anemia (hemoglobin: 6.1 g/dL) and mildly elevated creatinine (1.2 mg/dL), *β*-hCG of 722 IU/L and normal liver function tests. Abdominopelvic ultrasound and MRI showed an enlarged uterus (24 × 11 × 17 cm) with a large intrauterine heterogeneous mass that includes multiple discrete anechoic spaces consistent with complete mole (Figures 1 and 2). Moreover, the MRI revealed moderate right hydronephrosis with distal obstruction from the enlarged uterus. The ovaries and urinary bladder were unremarkable. Chest X-ray was normal. After receiving 2 units of packed red blood cells (PRBCs), she developed heavy vaginal bleeding requiring emergent dilation and evacuation (estimated blood loss: 1500 mL) and transfusion of 4 other units of PRBCs. Serum *β*-hCG level was >250,000 mIU/L on postoperative day 1. Histologic review confirmed complete hydatidiform mole. She had minimal vaginal bleeding in the postoperative period and her hemoglobin was 9.3 g/dL. On postoperative day 3, *β*-hCG dropped to 96,766 mIU/mL and creatinine became normal (0.5 mg/dL). Patient was discharged home after receiving an intramuscular injection of medroxyprogesterone acetate for contraception. Serial *β*-hCG values were performed every 1-2 weeks until reaching undetectable levels on postoperative day 120 and then at monthly intervals for an additional 6 months.

## 3. Discussion

In Europe and North and South America, hydatidiform moles are observed in approximately 1 in 1,000 pregnancies [5]. The prevalence is 5- to 15-fold higher in East Asia [5]. The karyotype of complete moles is usually 46,XX; the chromosomes derive completely from the father as a complete mole likely results from the fertilization of anuclear empty ovum by a haploid sperm that duplicates its own chromosomes after meiosis [6, 7]. Classical clinical signs of hydatidiform mole at diagnosis were vaginal bleeding, disproportionate increase in the size of the uterus, an abnormally high level of *β*-hCG for gestational age, hyperemesis gravidarum, cystic enlargement of the ovaries, and eventually hyperthyroidism and pregnancy-induced hypertension [8]. The widespread use of ultrasonography in early gestation and the enhanced accuracy of *β*-hCG assays have led to earlier diagnosis of molar pregnancy and subsequently changed its classical clinical presentation [9, 10]. An analysis of 113 cases of hydatidiform mole diagnosed in China between 1989 and 2006 showed that vaginal bleeding remained the most common presenting symptom (83.2%) followed by excessive uterine size (46%) [9]. However, the overall incidence of vaginal bleeding and preeclampsia were lower compared to historic data [9]. A review of the clinical presentation of 189 patients with hydatidiform mole diagnosed in Italy between 1992 and 2004 revealed that vaginal bleeding, which remained the most common symptom, became less frequent compared to those diagnosed between 1977 and 1985 (51% versus 74%, resp.; *P* < 0.0001) [10]. Similarly, the incidences of disproportionate enlargement of the uterus and presence of bilateral ovarian cysts have decreased with the advancement in technology and the earlier diagnosis [10]. Our patient presented with a 34-week size uterus compressing the right ureter leading to moderate right hydronephrosis and elevated serum creatinine level. The evacuation of the molar pregnancy relieved the ureteral obstruction and subsequently normalized the creatinine value. It is important to note that the patient had, in addition to the enlarged uterus, severe anemia with no vaginal bleeding. This can be explained by the accumulation of blood inside the uterine cavity causing the uterus to enlarge quickly and leading to the abrupt onset of heavy vaginal bleeding while she was in the hospital. This possibility is supported by the fast enlargement of the uterus combined with the lack of other causes of acute severe anemia. Therefore, acute anemia in similar cases of molar pregnancy should be cautiously interpreted, as heavy vaginal bleeding will likely occur spontaneously or during the evacuation procedure.

Complete molar pregnancies are often correctly diagnosed by ultrasound especially with the presence of characteristic placental features such as cystic changes and overt masses [11]. The ultrasound was very helpful in making the diagnosis of our patient after detecting a large intrauterine heterogeneous mass encompassing multiple anechoic spaces. The diagnosis was then confirmed by the histologic evaluation of the evacuated tissues.

Patients with complete mole usually have abnormally elevated *β*-hCG reaching greater than 100,000 mIU/L in approximately half of cases [3]. It is interesting to note that our patient initially had a relatively low *β*-hCG level for a complete mole, which may sometimes lead to a delay in the diagnosis. However, the fact that *β*-hCG level was much higher after evacuation suggests that the initial measurement was falsely low owing to the “hook effect” [12]. The extremely elevated *β*-hCG levels, usually above 500,000 mIU/L, can saturate both the immobilized capture antibodies and the free tracer antibodies. Thus, it can prevent the sandwich formation necessary for a positive test result, leading to either falsely low serum *β*-hCG test or falsely negative urine pregnancy test [12, 13]. Dilution of the sample to reduce the amount of *β*-hCG tested is advised to overcome this limitation. In our case, molar pregnancy evacuation reduced serum *β*-hCG concentrations leading to more accurate measurements.

The preferred treatment for molar pregnancy is suction dilation and evacuation [2]. Intravenous oxytocin following cervical dilation is recommended to decrease the risk of uterine atony. An important sequela is gestational trophoblastic neoplasia, which develops in 6–32% of complete moles. It is therefore recommended to monitor serum *β*-hCG levels every 1-2 weeks until reaching undetectable values, after which monthly measurements are required for an additional 6 months [2]. A reliable hormonal contraception is also needed until completing *β*-hCG monitoring.

In conclusion, complete molar pregnancy can present with an enlarged uterus compressing adjacent organs and severe anemia with no vaginal bleeding owing to the accumulation of blood in the uterine cavity. A low serum *β*-hCG level or negative urine pregnancy test in the setting of suspected complete mole should prompt repeating the measurement on diluted sample to prevent the “hook effect.”
